# Undetected Intracellular Immunoglobulin Light Chains in Aberrant Plasma Cells of Multiple Myeloma by Monoclonal Antibody‐Based Flow Cytometry: A Question Raised by a Single Case

**DOI:** 10.1155/jimr/2425288

**Published:** 2026-11-05

**Authors:** Jinyun Wang, Lulu Chen, Jie Xu, Yanchun Li, Ping Zhang

**Affiliations:** ^1^ Department of Clinical Laboratory Ninghai First Hospital Ninghai Zhejiang 315600 China; ^2^ Department of Clinical Laboratory Affiliated Hangzhou First People’s Hospital School of Medicine Westlake University Hangzhou Zhejiang 310006 China westlake.edu.cn; ^3^ College of Pharmacy Hangzhou Normal University Hangzhou Zhejiang 311121 China hznu.edu.cn; ^4^ Department of Pharmacy Sir Run Run Shaw Hospital School of Medicine Zhejiang University Hangzhou Zhejiang 310016 China zju.edu.cn

**Keywords:** flow cytometry, immunoglobulin light chain, multiple myeloma, polyclonal antibody

## Abstract

This study presents a clinical case demonstrating the limitations of monoclonal antibodies (MAbs) in flow cytometric immunophenotyping for clonal disorders. Following standardized protocols, we performed surface antigen and intracellular light chain staining, using SSC/FSC gating with CD45/SSC and CD138/CD38 combinations to identify plasma cell populations. Flow cytometry analysis revealed abnormal plasma cell immunophenotypes (CD138^+^CD38^+^CD56^+^CD45^−^CD19^−^ with partial CD117 expression) in a patient with multiple myeloma (MM), in which the MAb assay showed no detectable cytoplasmic κ or λ staining. Normal plasma cells showed characteristic CD138^+^CD38^+^CD45^+^CD19^+^CD56^−^ expression with polyclonal light chain distribution (*κ*/*λ* ratio 1.43). These findings were further supported by bone marrow morphology showing immature plasma cells and immunofixation electrophoresis confirming an IgG‐λ monoclonal protein. Our findings indicate a risk of false‐negative results in intracellular light‐chain detection using MAbs. Although polyclonal antibodies (PAbs) have been reported to potentially overcome the limitations of monoclonal reagents, unfortunately, no PAbs were available to test this in our case. Finally, this study systematically analyzes the reasons for the discordance between flow cytometric detection based on MAbs and other detection methods, and suggests that clonal assessment in MM requires integrating evidence from multiple detection methods for orthogonal validation to improve diagnostic accuracy.

## 1. Introduction

Multiple myeloma (MM) is characterized by the abnormal proliferation of clonal malignant plasma cells (myeloma cells) in the bone marrow, accompanied by an increase in monoclonal immunoglobulins [1]. This disease is more common in the elderly and has a variety of clinical manifestations. The main symptoms include the proliferation, infiltration, and destruction of bone tissue by myeloma cells, as well as the impact on other extramedullary tissues [2]. Patients may experience bone pain, pathological fractures, anemia, and bleeding. Additionally, due to the large number of monoclonal immunoglobulins produced by myeloma cells, patients may also suffer from infections, hypercalcemia, renal disorders, hyperviscosity syndrome, and amyloidosis [3]. Early diagnosis and prompt treatment are crucial for controlling the progression of the disease and improving the patient’s prognosis.

At present, in addition to the patient’s clinical symptoms, a variety of testing methods are available to assist in the diagnosis of MM. These include bone marrow biopsy and cytomorphological examination of bone marrow smears, serum protein electrophoresis and immunofixation electrophoresis, fluorescence in situ hybridization (FISH) technology, and quantitative detection of immunoglobulin and serum/urine light chain levels [4, 5]. Bone marrow cytomorphological examination and immunoelectrophoresis are the cornerstone diagnostic techniques widely implemented. However, the focal distribution of MM within the bone marrow and variations in puncture sites can make it difficult to detect myeloma cells in some cases through morphological assessment alone. Moreover, distinguishing myeloma cells from reactive and normal plasma cells based on morphology is challenging and can be influenced by the subjectivity of the physician, which heightens the risk of misdiagnosis or missed diagnosis in clinical settings. Immunofixation electrophoresis, which combines electrophoretic separation with diffusion, is a highly sensitive and specific assay. It not only detects monoclonal proteins (M‐proteins) with great sensitivity but also accurately classifies their types [6]. However, in cases of nonsecretory myeloma patients, M‐proteins are often undetectable by this method. Quantitative immunoglobulin testing and the assessment of serum and urine light‐chain levels offer the advantages of testing speed and accuracy. However, these methods do not indicate the presence of M‐proteins and also have limited sensitivity for diagnosing MM [7]. In summary, neither bone marrow cytomorphology nor immunofixation electrophoresis alone can be considered the gold standard for diagnosing MM. To avoid misdiagnosis and missed diagnosis and ensure a more accurate and comprehensive assessment, a combination of various diagnostic approaches should be employed for the clinical diagnosis of MM [8, 9].

Multiparameter flow cytometric immunophenotypic analysis plays an irreplaceable role in the diagnosis, treatment guidance, and prognosis assessment of MM and can be used for the analysis of minimal residual disease [10–12]. Within the flow cytometric testing framework, the clonality assessment of cytoplasmic immunoglobulin light chains represents the central step in distinguishing reactive from neoplastic plasma cells and relies principally on fluorochrome‐conjugated antibody reagents. Monoclonal antibodies (MAbs), produced by a single B‐cell clone, recognize only a single epitope on the antigen molecule, and have served as the reagent of choice in flow cytometry [13]. In contrast, polyclonal antibodies (PAbs), generated by multiple B‐cell clones, simultaneously recognize several distinct epitopes on the antigen molecule [14]. In current clinical practice, the majority of MM detection protocols rely on MAbs, with insufficient attention directed toward the value of PAbs. In this report, we present a case of MM in which aberrant plasma cells showed no detectable cytoplasmic κ/λ staining by a standardized MAb‐based flow cytometric assay, despite serological and morphological evidence of an IgG‐λ monoclonal plasma cell proliferation. We describe the immunophenotypic findings in detail and discuss the possible explanations for this phenomenon, as well as the rationale and benefits for validating complementary reagents such as PAbs in future comparative studies.

## 2. Materials and Methods

### 2.1. Clinical Data

The patient was a 74‐year‐old female who presented with fatigue and discomfort along with right shoulder joint pain for over 7 months without any apparent cause; there was no nausea or vomiting, fever, jaundice, rash, petechiae, hematemesis, or melena; a routine blood test indicated mild anemia. Bone marrow cytomorphological examination showed an increased proportion of plasma cells with easily observable immature plasma cells; serum immunofixation electrophoresis revealed IgG, λ type, with the presence of light chain λ type M‐protein; according to the diagnostic criteria in the “Chinese Guidelines for the Diagnosis and Treatment of Multiple Myeloma”, a diagnosis of MM was confirmed.

### 2.2. Equipment and Reagents for Flow Cytometry

A BD FACSCanto II flow cytometer (BD Biosciences, San Jose, CA, USA) was used. MAbs included CD138, CD38, CD19, CD56, CD45, κ, λ, CD27, CD28, CD117, and CD20, with detailed information provided in Table 1. Phosphate‐buffered saline (PBS), red blood cell lysing solution (BD Multitest IMK lysing solution), and the fixative and permeabilization reagent (BD IntraSure kit) required for intracellular antigen staining were all sourced from the American BD Company.

**Table 1:** Details of antibodies commonly used in flow cytometry for the diagnosis of MM.

Antigen	Fluorochrome	Clone	Catalog number
κ	FITC	TB 28‐2	BD652813
λ	PE	1‐155‐2	BD652812
CD56	ECD	N901	Beckman, C41152, A82943
CD27	PC7	1A4CD27	Beckman, B49205, A54823
CD19	PerCP	SJ25C1	BD652826
CD138	APC	MI15	BD347193
CD38	Alexa Fluor 700	HIT2	BD560676
CD45	APC‐CY7	2D1	BD663488
CD20	FITC	L27	BD652808
CD45	PerCP	2D1	BD664934
CD117	PE‐CY7	104D2	BD663491
CD28	APC‐H7	CD28.2	BD561368

### 2.3. Flow Cytometer Analysis of Plasma Cell Immunophenotype

Flow cytometry analysis of circulating plasma cells in peripheral blood has proven to be an important assessment method for MM [15, 16]. It is less invasive, more accessible, and better tolerated by patients than bone marrow aspiration. The patient’s EDTA‐anticoagulated peripheral blood (1 mL) was added to PBS for washing. After centrifugation at 500 *g* for 5 min, the supernatant was carefully discarded, and this washing process was repeated three times. The sample was divided into two tubes for staining: tube 1 (FIRST1) was stained with CD19, CD38, CD45, CD27, CD56, CD138, and cytoplasmic κ and λ; tube 2 (FIRST2) was stained with CD28, CD20, and CD117. About 100 μL of the washed blood sample was incubated with fluorescently labeled antibodies in a flow cytometry tube at room temperature. After incubation in the dark for 15 min, 500 μL of red blood cell lysing solution was added, mixed well by vortexing, and incubated at room temperature for 10 min again. Then, 2 mL of PBS was added, vortexed, and centrifuged at 800 *g* for 5 min. The washed cells were used for analysis by flow cytometry. For intracellular antigen staining, after incubation with surface antigen antibodies, the labeled cells were incubated with 100 μL Solution A (fixation) for 5 min, and then red blood cell lysing solution was added and incubated in the dark for 10 min. After centrifugation at 800 *g* for 5 min, the cells were incubated with 50 μL Solution B (permeabilization) and the intracellular antigen antibodies (immunoglobulin light chains κ and λ) were kept at room temperature in the dark for 15 min. After being washed with PBS, the cells were analyzed by flow cytometry. Flow cytometric data acquisition and analysis were performed on a BD FACS Canto II flow cytometer and FACS Diva software (version 8.0.2), respectively. Data were acquired with an FSC threshold of 5000, and fluorescence signals were collected on a 4‐log decade scale. A total of 528,000 events were acquired, with a detection limit of approximately 1 in 4.5 × 10^5^ analyzed cells. Daily quality control was conducted using BD CST beads. The flow cytometer underwent a precise calibration for fluorescence, ensuring that all parameters were meticulously configured and the fluorescence compensation was finely tuned to optimal levels before the initiation of the detection process.

The gating strategy for identifying plasma cells by flow cytometry is as follows. First, the forward scatter‐area (FSC‐A) versus forward scatter‐height (FSC‐H) was used to exclude cell doublets and aggregates for downstream analysis, ensuring that each event represents an individual cell. Then, FSC‐A versus CD45 (leukocyte common antigen) was applied to exclude debris. For plasma cell identification, CD138‐positive cells, a specific plasma cell marker, were identified regardless of CD38 dim expression (P8). The absolute number of abnormal plasma cells was calculated as the percentage of gated leukocytes (FSC‐A vs. CD45) multiplied by the total white blood cell count (0.053% of gated leukocytes, corresponding to approximately1.8 cells/μL in this patient). Side scatter‐area (SSC‐A) versus CD45 plot (P9 gate) was further applied to confirm their scatter properties, and CD45 expression intensity was used to distinguish between normal (shown in pink) and abnormal plasma cells (shown in red). Furthermore, the expression of additional aberrant markers often expressed on myeloma cells was assessed on these distinct abnormal plasma cells identified by the CD45‐negative/CD138‐positive population. The last is the critical clonality assessment through cytoplasmic light chain restriction (κ vs. λ) analysis. The abnormal plasma cells lacked detectable staining with the tested MAb assay, while normal plasma cells (pink) and polyclonal B cells (light blue) show a balanced κ/λ ratio, which was also used as an internal quality control. More detailed information can be found in the original FCS file in the Supporting Information.

### 2.4. Serum Immunofixation Electrophoresis

The separated venous serum samples were meticulously prepared and subjected to agarose gel electrophoresis. Initially, 10 μL of diluted serum samples was applied to five lanes for electrophoresis. Following the completion of electrophoresis, the protein fixative was sequentially added to each lane, followed by the introduction of five corresponding antibodies to observe the migration patterns of the proteins. Ultimately, a detailed interpretation of the electrophoretic patterns was conducted to accurately analyze the protein components present in the serum samples.

### 2.5. Cytomorphological Examination of Bone Marrow

A collected 0.2 mL of heparin‐anticoagulated bone marrow aspirate was smeared onto slides and air‐dried at room temperature. Following this, the slides were stained with the Wright–Giemsa stain for 15 min, rinsed under running water, and allowed to dry. Subsequently, a skilled and dedicated bone marrow cytomorphologist performed the microscopic examination, meticulously observing the morphology and proportion of myeloma cells.

### 2.6. Other Laboratory Tests

The quantitation of serum immunoglobulins (IgG/IgA/IgM) and the light chains (κ and λ) was performed using an immunoturbidimetric assay; the experimental process was strictly carried out according to the standard operating procedures of the instrument and reagents, and all test items were under quality control.

## 3. Results

### 3.1. Flow Cytometry Detection of Plasma Cell Immunophenotype

As shown in Figure 1, utilizing a gating strategy for flow cytometry analysis based on FSC (forward scatter), SSC (side scatter), CD45, CD138, and CD38, the immunophenotypic profile of abnormal plasma cells in an MM patient was characterized as follows: CD138 and CD56 were positively expressed, CD38 was dimly positive, CD45, CD19, and CD27 were negative, CD117 was partially positive, and CD28 was occasionally positive. Notably, abnormal plasma cells lacked detectable staining with the tested MAb assay. In contrast, the immunophenotype of normal plasma cells was defined by the positive expression of CD138, CD38, CD45, CD19, and CD27, the negative expression of CD56, CD117, and CD28, and a polyclonal expression of intracellular light chains, with a κ/λ ratio of approximately 1.43. Additionally, under the same flow cytometry detection protocol using monoclonal immunoglobulin light‐chain antibodies, B cells also displayed a normal polyclonal light‐chain expression pattern. This indicates that the light chains expressed in the patient’s abnormal plasma cells are not recognized by conventional MAbs.

**Figure 1:**
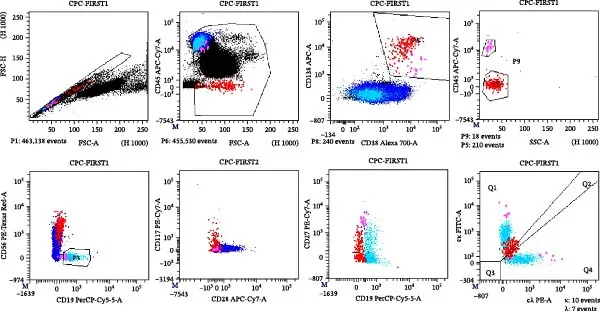
Immunophenotypic analysis of the plasma cells in blood samples through flow cytometry. Pink dots indicate normal plasma cells with polyclonal expression of intracellular light chains, and the ratio of intracellular light chain of κ to λ is 1.43, while the red dots indicate abnormal plasma cells that lacked detectable staining with the tested MAb assay. Sky blue is the B cells used as the internal control. Data were obtained from a single peripheral blood sample from one patient, and the analysis was performed once.

### 3.2. Immunofixation Electrophoresis of the Serum

The results of serum immunofixation electrophoresis analysis showed abnormal monoclonal bands in the IgG and λ lanes, indicating that the type of immunoglobulin secreted by the patient’s plasma cells is monoclonal IgG‐λ type (Figure 2).

**Figure 2:**
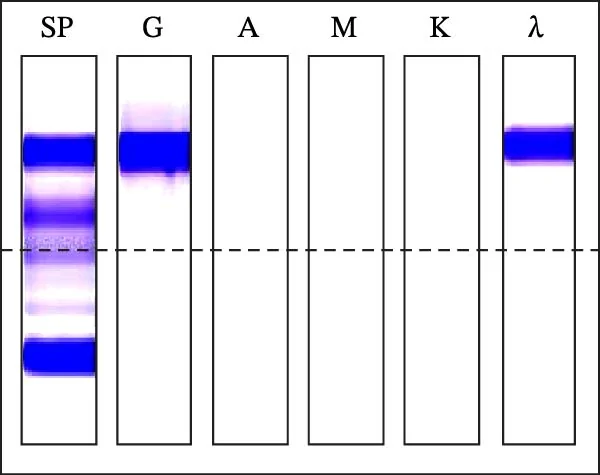
Results of the patient’s serum from immunofixation electrophoresis. SP is the standard; G indicates immunoglobulin G; A indicates immunoglobulin A; M indicates immunoglobulin M; K indicates the *κ* light chain of immunoglobulin; and λ indicates the *λ* light chain of immunoglobulin.

### 3.3. Morphological Analysis of Bone Marrow Smear

The bone marrow cytomorphological examination revealed a significantly elevated proportion of plasma cells, with plasmablasts and immature plasma cells comprising an impressive 47.5% (Figure 3). The distinctive morphological characteristics of these cells are as follows: they display a wide range of sizes, highlighting pronounced pleomorphism. The cytoplasm is moderately abundant, presenting a deep blue or flame hue, occasionally containing sparse granules but commonly lacking a perinuclear clear zone. The nuclei are often eccentric, with instances of binucleation or multinucleation. The chromatin is delicately arranged in a loose, reticular pattern, and nucleoli are typically present in numbers ranging from one to two. These findings point to aberrant proliferation and differentiation of plasma cells within the bone marrow.

**Figure 3:**
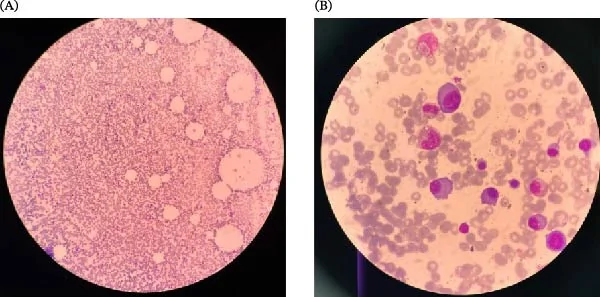
Wright–Giemsa staining results of a bone marrow smear. (A) The result at 100x magnification and (B) the result at 1000x magnification.

### 3.4. Routine Laboratory Examination

The results of other laboratory examinations and the reference interval are shown in Table 2. Data showed that the λ light chain in serum was significantly increased (22.4 g/L), together with an increase in immunoglobulin G and β2‐microglobulin. In addition, the serum free light chain κ (normal reference interval: 6.7–22.4 mg/L) was 6.22 mg/L; serum free light chain λ (normal reference interval: 8.3–27.0 mg/L) was 260 mg/L, far greater (10 times higher) than normal levels, and the ratio of free κ to λ (normal reference interval: 0.31–1.56) was 0.02. These results strongly indicate a diagnosis of MM with restricted expression of the λ light chain.

**Table 2:** Assessment of other clinical parameters from the patient’s blood and serum.

Clinical laboratory test	Result	Normal reference value	Unit
White blood cell count	3.4	3.5–9.5	× 10^9^/L
Red blood cell count	2.62	3.8–5.1	× 10^12^/L
Platelet count	173	125–315	× 10^9^/L
Hemoglobin	80	115–150	g/L
Reticulocyte	2.5%	0.5%–1.5%	—
Total protein	70.2	65.0–85.0	g/L
Albumin	34.3	40.5–55.0	g/L
Alanine transaminase (ALT)	15	7–40	U/L
Lactic dehydrogenase (LDH)	157	120–250	U/L
Urea	8.52	3.1–8.8	mmol/L
Creatinine	82	41–88	μmol/L
Calcium	2.18	2.0–2.6	mmol/L
Immunoglobulin G (IgG)	27.6	8.6–17.4	g/L
Immunoglobulin A (IgA)	<0.05	1.0–4.2	g/L
Immunoglobulin M (IgM)	0.10	0.5–2.8	g/L
*κ* light chains	0.97	6.3–13.5	g/L
*λ* light chains	22.4	3.1–7.2	g/L
*κ*/*λ*	0.04	1.53–3.29	—
Free *κ* light chains	6.22	6.7–22.4	mg/L
Free *λ* light chains	260.00	8.3–27.0	mg/L
Free *κ*/*λ*	0.02	0.31–1.56	—
β2‐microglobulin	3.1	1.01–1.73	mg/L
Ferritin	342.10	4.63–204.00	μg/L

## 4. Discussion

The survival rate of MM has improved substantially, largely attributable to the advent of novel therapeutic agents and more precise diagnostic strategies [2]. In addition to the conventional methods, such as bone marrow biopsy cytomorphology examination, immunofixation electrophoresis, and quantitative detection of immunoglobulins and light chain levels in blood and urine, flow cytometry has become an essential diagnostic tool for hematologic malignancies due to its advantages of rapid detection, multiparameter analysis, and high sensitivity [17, 18]. As the core reagents of the flow cytometry detection system, MAbs have become increasingly important in the diagnosis and treatment of MM [19]. However, in this case, the MAb‐based assay showed no detectable cytoplasmic light‐chain staining in aberrant plasma cells despite serological and morphological evidence of an IgG‐λ monoclonal proliferation. We regard this finding as an assay discordance that highlights a potential pitfall of MAb‐based light‐chain detection in MM.

In the routine multiparameter flow cytometric testing framework, CD38 and CD138 serve as the core markers for identifying and gating plasma cell populations. Normal plasma cells typically exhibit the immunophenotype CD38^+^CD138^+^CD45^+^CD19^+^, whereas aberrant plasma cells in the MM patient frequently display a CD45^−^/CD19^−^CD56^+^ profile [20–23]. The downregulation of CD27 and the heterogeneous CD38 expression also provide valuable diagnostic reference points in MM [24, 25]. The combined analysis of these immunophenotypic markers not only facilitates the distinction between normal and malignant clonal plasma cells but also establishes a robust technical foundation for MRD monitoring [23].

In addition to the expression pattern of surface antigens, flow cytometry is also important to identify the expression characteristics of intracellular light chains in plasma cells. Due to the isotype exclusion mechanism based on the rearrangement of immunoglobulin light chain genes, a single clone of plasma cells produces only one type of light chain. In healthy individuals, plasma cells are polyclonal and secrete a variety of immunoglobulins, which consist of a pair of light chains (κ and λ) and a pair of heavy chains. Therefore, normal plasma cells exhibit a polyclonal expression of light chains. However, upon malignant transformation of plasma cells, there is an abnormality in immunoglobulin synthesis, often characterized by the overproduction of a single type of light chain. The measurement of intracellular light chains in plasma cells is an important indicator for identifying abnormal plasma cells. Therefore, employing flow cytometric immunophenotyping, we can achieve an objective evaluation of the plasma cell phenotype, including its heterogeneity. This technique further allows for the precise measurement of the clonality and quantity of intracellular light chains. By integrating these plasma cell phenotypic characteristics, it becomes feasible to effectively differentiate myeloma cells from normal plasma cells.

Owing to their high specificity and lot‐to‐lot consistency, MAbs have long been regarded as the gold standard reagent for immunoglobulin light chain detection by flow cytometry. Nevertheless, some evidence indicates that MAbs harbor intrinsic deficiencies in detecting cytoplasmic light chains in clonal plasma cells from certain MM patients. Van Velzen et al. [26] reported a typical case of MM in which the epitope recognized by the monoclonal anti‐λ light chain antibody was not expressed on the pathological immunoglobulin. Upon switching to PAb, λ light chains were clearly detected [26]. A study encompassing 564 B‐cell non‐Hodgkin lymphoma specimens found a discordance rate of 9.2% between MAb‐ and PAb‐based light chain expression detection. Although no antibody showed superior ability, the combined use of PAb and MAb increased the accuracy of light‐chain detection [27]. These literature reports are similar to the results of this study, suggesting a risk of false‐negative results in MAb‐based assays in a subset of patients.

Several mechanisms may underlie the false‐negative results with MAbs. Immunoglobulin light chains in neoplastic plasma cells may undergo aberrant folding, glycosylation modifications, or association with other molecules, thereby occluding the single epitope targeted by the MAb [28]. The immunoglobulin gene variable regions in MM cells may undergo high‐frequency mutations, resulting in the loss of the specific epitope recognized by the MAb. Although the use of a standardized commercial kit and the successful detection of surface markers in this study reduce the likelihood of technical artifacts, a contributory role of technical confounders in the false‐negative MAb‐based light chain detection cannot be entirely excluded.

Previous studies have suggested that PAbs may compensate for certain limitations of MAbs. Van Velzen et al. [29] demonstrated in follicular lymphoma cases that the use of MAbs to detect the light chain of B‐cell membrane‐bound immunoglobulins may lead to false‐negative results, while PAbs were able to compensate for this misdiagnosis. PAbs offer broader epitope coverage, higher sensitivity for low‐abundance targets, and greater tolerance of diverse molecular conformations by binding multiple distinct epitopes on the same antigen [28]. Simultaneous binding of several antibody molecules also generates a stronger fluorescence signal, further improving detectability, and the multi‐epitope nature of PAbs renders their binding less susceptible to single‐epitope loss caused by somatic mutations in MM cells. Nevertheless, PAbs also have their limitations: their broader reactivity may increase background and nonspecific staining; their specificity is lower than that of MAbs; and batch‐to‐batch variability is inherent to their production. Accordingly, PAbs should complement rather than replace MAbs, and the relative performance of the two reagent classes can only be established through a systematic head‐to‐head comparison.

Notably, plasma‐cell clones are biologically heterogeneous, and their immunoglobulin production, intracellular processing, and surface phenotype may vary across anatomical compartments and microenvironmental niches [30]. The staining pattern observed in our case is consistent with this possibility. Besides, a recent spatial profiling of matched bone marrow and extramedullary myeloma revealed markedly divergent antigen expression between sites, including CD38 downregulation in extramedullary plasma cells, illustrating that microenvironmental selective pressures can shape phenotypically distinct subclones [31]. Single‐site sampling may therefore underestimate divergent subclones in spatially heterogeneous disease. For future diagnostic practice, this supports a broader principle: clonality in MM should be assessed orthogonally, integrating flow cytometry with serological, morphological, and molecular evidence so that a negative result from a single reagent or a single compartment does not prematurely exclude biologically relevant clonal plasma cells.

However, this study has certain limitations. First, this study is based on the observation of a single case and has a limited sample size, so the reported phenomena cannot yet be generalized to the broader population of MM patients. Second, because no residual diagnostic bone marrow material was available, the cytoplasmic light‐chain assay could not be repeated on the marrow aspirate, and the analysis was performed on peripheral blood instead. Peripheral blood may not fully reflect the clonal composition of the bone marrow plasma cell compartment. Third, due to the unavailability of PAbs, we were unable to further validate the proposed hypothesis. Future studies should systematically evaluate the immunophenotypic characteristics of MM patient cells in a larger sample cohort, combined with direct comparison of monoclonal and polyclonal light chain antibodies. Such studies will help to more accurately assess the immune characteristics of such clonal diseases and elucidate their heterogeneity and pathological complexity, thus providing a basis for establishing a more precise diagnostic framework.

In conclusion, our case raises a discordance between flow cytometric non‐detection of cytoplasmic light chains and independent serological and morphological evidence of an IgG‐λ clone in a patient with MM. While this observation highlights a potential pitfall of MAb‐based assays, it does not establish an intrinsic failure of monoclonal reagents. As a complementary approach, PAbs may be able to compensate for the limitations of MAbs in selected cases, but this potential benefit requires direct validation in comparative studies. More broadly, clonality assessment in MM is best achieved through orthogonal verification that integrates evidence from multiple modalities, thereby improving the diagnostic accuracy.

## Author Contributions

**Jinyun Wang**: writing – original draft. **Lulu Chen:** writing – original draft, software. **Jie Xu**: data curation. **Yanchun Li**: writing – reviewing and editing, resources, visualization. **Ping Zhang**: conceptualization, data curation, resources, supervision.

## Funding

This work was supported by the Zhejiang Medical and Health Science and Technology Project (Grants 2025KY125, 2025KY230, and 2023RC228).

## Ethics Statement

All procedures followed were in accordance with the ethical standards of the responsible committee on human experimentation (institutional and national) and with the Helsinki Declaration of 1964 and later versions. Informed consent to be included in the study, or the equivalent, was obtained from the patient. This study was approved by the Ethical Committee of the Hangzhou First People’s Hospital (2025ZN030‐1).

## Conflicts of Interest

The authors declare no conflicts of interest.

## Supporting Information

Additional supporting information can be found online in the Supporting Information section.

## Supporting information


**Supporting Information** Raw flow cytometry data (FCS) for both staining tubes and the FACSDiva experiment file (XML) documenting the complete gating hierarchy, per‐gate event counts, and acquisition settings, enabling independent verification of the analysis.

## Data Availability

The data that support the findings of this study are available in the Supporting Information of this article.
